# Coincidence energy spectra due to the intrinsic radioactivity of LYSO scintillation crystals

**DOI:** 10.1186/s40658-020-00291-1

**Published:** 2020-04-15

**Authors:** Francisco Eduardo Enríquez-Mier-y-Terán, Ana Saret Ortega-Galindo, Tirso Murrieta-Rodríguez, Mercedes Rodríguez-Villafuerte, Arnulfo Martínez-Dávalos, Héctor Alva-Sánchez

**Affiliations:** 1 Facultad de Ciencias, Universidad Nacional Autónoma de México, 04510 Mexico City, Mexico; 2 Instituto de Física, Universidad Nacional Autónoma de México, A.P. 20-364, 01000 Mexico City, Mexico

**Keywords:** LYSO, Intrinsic radioactivity, Coincidence detection, Monte Carlo simulations, GATE, PET detector

## Abstract

**Background:**

Lutetium oxyorthosilicate or lutetium yttrium oxyorthosilicate (LYSO) scintillation crystals used in most current PET scanner detectors contain ^176^Lu, which decays by beta emission to excited states of ^176^Hf accompanied by the emission of prompt gamma rays or internal conversion electrons. This intrinsic radioactivity can be self-detected in singles mode as a constant background signal that has an energy spectrum whose structure has been explained previously. In this work, we studied the energy spectrum due to the intrinsic radioactivity of LYSO scintillation crystals of two opposing detectors working in coincidence mode. The investigation included experimental data, Monte Carlo simulations and an analytical model.

**Results:**

The structure of the energy spectrum was completely understood and is the result of the self-detection of beta particles from ^176^Lu in one crystal and the detection of one or more prompt gamma rays detected in coincidence by the opposing crystal. The most probable coincidence detection involves the gamma rays of 202 and 307 keV, which result in two narrow photopeaks, superimposed on a continuous energy distribution due to the beta particle energy deposition. The relative intensities of the gamma ray peaks depend on crystal size and detector separation distance, as is explained by the analytical model and verified through the Monte Carlo simulations and experiments.

**Conclusions:**

The analytical model used in this work accurately explains the general features of the coincidence energy spectrum due to the presence of ^176^Lu in the scintillation crystals, as observed experimentally and with Monte Carlo simulations. This work will be useful to those research studies aimed at using the intrinsic radioactivity of LYSO crystals for transmission scans and detector calibration in coincidence mode.

## Introduction

The discovery of cerium-doped lutetium oxyorthosilicate (LSO) by Melcher and Schweitzer [1] gave rise to a series of advances in positron emission tomography (PET) imaging. This lutetium-based scintillation crystal, and the later-developed lutetium yttrium oxyorthosilicate (LYSO), offered a fast decay time (40 ns) and a high light output (30 photons/keV), with a similar mean free path value for annihilation photons (11.4 mm) compared to bismuth germanate (BGO) scintillation crystals (10.4 mm) [2]. All these properties have permitted the development of new PET scanners with improved spatial and energy resolutions, with high-count rate performance and the implementation of other technological advances such as time-of-flight (TOF) PET.

**Fig. 1:**
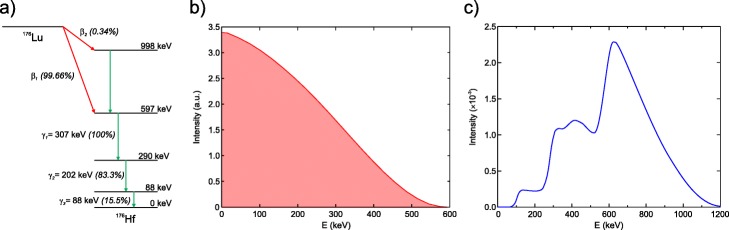
**a** Simplified decay scheme of ^176^Lu, **b** β_1_-particle energy spectrum (data taken from [4]), and **c** experimental singles energy spectrum due the intrinsic radiation of a LYSO crystal (57.4 × 57.4 × 10 mm^3^)

Among the different physical properties that these lutetium-based scintillation crystals present, there is one that has been of special interest since its discovery: its intrinsic radioactivity due to the presence of ^176^Lu (2.6%) in natural lutetium [3]. ^176^Lu decays via β^−^ (*E*_max_ = 593 keV) to ^176^Hf followed by the emission of prompt gamma rays (γ_1_, γ_2_ and γ_3_ with energies of 307, 202 and 88 keV, respectively) or related internal conversion (IC) processes competing with the emission of γ_2_ or γ_3_, as a result of the isomeric transitions of the excited states of ^176^Hf. The ^176^Lu simplified decay scheme and the continuous β^−^ energy spectrum are shown in Fig. 1a and b, respectively. The intrinsic radioactivity accounts for approximately 300 Bq/cm^3^, which when self-detected produces a rate of events that cannot be disregarded. An example of a background spectrum in singles mode for a large (57.4 × 57.4 × 10 mm^3^) monolithic crystal is included in Fig. 1c.

In coincidence mode, the intrinsic radioactivity in LSO/LYSO-based PET scanners produces the detection of true coincidence and multiple coincidence events during a study [5]. The number of false lines of response arising from these events, which depends on the number of scintillation crystals and the scanner architecture, can be reduced by increasing the lower level discriminator [6], narrowing the energy window of the scanner [7] or by reducing the coincidence time window. Yoshida et al*.* [7] developed a reduction method for the intrinsic random coincidences that uses TOF capabilities of modern PET scanners and Monte Carlo simulations for multiple coincidence information. In many cases, if the activity of the administered radiopharmaceutical is large enough, the events due to ^176^Lu may be neglected in a typical clinical PET scanner, but that may not be the case for small-animal imaging protocols [8], particularly for those aimed at cell trafficking studies and gene expression imaging [9] and in organ-specific positron emission imaging probes.

In modern scanner architectures, like the EXPLORER, a novel high sensitivity total-body system [10] that involves the use of several times the LYSO crystal volume of standard PET scanners, the intrinsic radioactivity might represent a concern especially when reducing the injected dose to the patients below 1 mCi.

Despite the drawbacks that in certain scenarios this intrinsic radioactivity might represent, several authors have proposed its use as a permanent built-in “field-flood” source [11]. Knoess et al. [12] and Rothfuss et al. [13] measured detector-block sensitivities and crystal energy spectra using the background radioactivity of LSO crystals, suggesting that this intrinsic background signal can be used for daily quality checks in LSO/LYSO-based PET scanners. Conti et al. [14], using a conventional ^68^Ge external source and the LSO intrinsic radiation, performed energy calibrations of a PET system, establishing that the positions of the ^176^Lu peaks in the energy spectrum can be used for energy calibration without any external source and used in a daily quality control protocol of the scanner. Moreover, with the development of state-of-the-art silicon photomultipliers (SiPMs), the implementation of TOF PET imaging has been a major advance in the field.

Rothfuss et al. [15] demonstrated that using the LSO intrinsic radioactivity and TOF information, it is possible to obtain transmission images and, consequently, perform attenuation corrections to the PET images without the need for a computed tomography scan. This could reduce artifacts arising from patient movement or errors in image registration and can be effectively used in multi-ring scanners containing a large number of LYSO crystals [16].

Using the background radiation as a “field-flood” source for detector calibration and attenuation correction requires a complete characterization and understanding of the ^176^Lu intrinsic energy spectrum in singles and coincidence mode. In singles mode, this intrinsic energy spectrum has been previously reported [17–21]. In 2010, Goertzen et al. [22] developed a method for estimating the energy spectrum of coincidence events in a PET system and compared it with measurements and Monte Carlo simulations using GATE (Geant4 Application for Tomographic Emission) [23]. A more detailed model of ^176^Lu in GATE was further reported by McIntosh et al. [24]. Other uses of the LYSO intrinsic radiation have been put forward, such as the crystal identification in dual-layer-offset detectors using both singles and coincidence data acquisitions [25].

In a recent work, we proposed a more complete explanation of the energy spectrum in singles mode as a function of the crystal size using an analytical model [26]. Later, a further validation of these analytical calculations was performed by Enríquez-Mier-y-Terán et al. [27] through Monte Carlo simulations using GATE v.8.1, exhibiting a notable agreement between the experiments, calculations and simulations.

The main purpose of this work is to understand the physical processes to explain the structure of the energy spectrum arising from two opposing LYSO crystals, operating in coincidence mode, due to the ^176^Lu intrinsic radioactivity. To this purpose, we present a model that considers the probabilities of decay, emission and absorption of the ionizing radiation involved and their relative contributions to the spectrum. We extended the analytical model used to unravel the structure of the background energy spectrum in singles mode, to explain the structure of the energy spectrum of two opposing LYSO crystals in coincidence mode as a function of crystal separation distance. We compared the analytical predictions to experimental data and Monte Carlo simulations using GATE v8.1.

## Methods

### Analytical calculations

The analytical calculations to predict the shape of the coincidence energy spectrum were carried out considering two opposing LYSO scintillation crystals of size 57.4 × 57.4 × 10 mm^3^, with the large 57.4 × 57.4 mm^2^ side facing each other directly. The calculations assumed that the ^176^Lu beta decays (and their corresponding γ-rays and IC electrons cascade) occurred in one crystal and that the coincidence events were the result of one, two or all three γ-rays, from the isomeric transitions of the excited states of the ^176^Hf, detected in the opposing crystal. In other words, one crystal was used as the source crystal of β^−^, γ-rays and IC electrons while the twin crystal served only as a γ-ray detector crystal.

For every β^−^ decay of ^176^Lu, isomeric transitions of ^176^Hf occur (γ-rays and/or IC electrons). The total energy deposited in the crystal is the sum of the β^−^ kinetic energy and that deposited by IC electrons and/or γ-ray interactions. In a similar manner as in [26], a shift in the β^−^ energy spectra (the translation of the complete shape of the continuous spectrum to the right, i.e., to a higher energy value) is done whenever there is energy deposited by the isomeric transition radiation. The energy value to which the β^−^ spectrum is shifted corresponds to the energy deposited by one or two γ-rays (or internal conversion electrons) in the source crystal.

Note that the combination of self-detecting all three γ-rays in the source crystal is not allowed since at least one γ-ray needs to escape from this crystal and reach the detector crystal for a coincidence event to be registered. Every time the ^176^Lu β^−^ energy spectrum is shifted, it is also normalized so that the area under the curve reflects the probability of occurrence of each particular combination, taking into consideration the probability values of the γ-rays emissions and the IC processes, and assuming that all β^−^ particles and IC electrons deposit all their energy within the crystal, as described in [26]. To account for the probabilities of the γ-rays interacting (or not) in the source and/or in the detector crystal, Monte Carlo simulations of monoenergetic photons were performed as described in the “Simulation of monoenergetic photons in singles mode for the analytical model” section.

The coincidence energy spectrum was calculated as the weighted sum of the β^−^ spectra shifted by the energy deposited due to the electromagnetic cascade interactions in the source crystal during the ^176^Lu decay, plus the weighted contribution of photopeaks produced by the photoelectric absorption combinations of γ_1_, γ_2_ and γ_3_ in the detector crystal. The weighted values were obtained from the ^176^Lu decay probabilities (Fig. 1a) and Monte Carlo simulations of monoenergetic gamma rays absorption probabilities in the source and detector crystals for a given crystal geometry and separation distance *d*.

The calculations considered only true coincidences, that is, events arising from the β^−^ decay in the source crystal and the detection of, at least, one gamma ray from the same decay process in the detector crystal. Random coincidences, in which β^−^ decay occurs in both LYSO crystals within the coincidence time window, were not included in the model. However, the rate of these rare events is low, as it was corroborated both experimentally and in the Monte Carlo simulations.

**Fig. 2:**
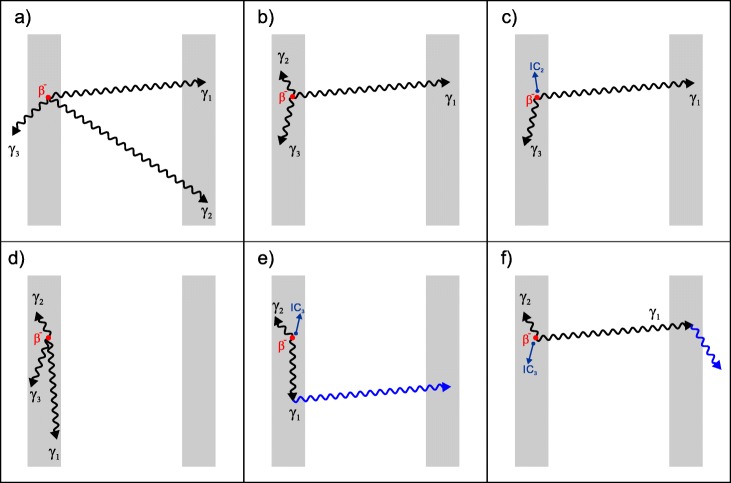
Examples of coincidence events between two opposing crystals due to the natural radioactivity of LYSO scintillation crystals. The crystal on the left is the source crystal, while its opposite is the detector crystal. The top and bottom rows show examples of events considered and not considered, respectively, in the analytical model

Figure 2 includes some examples of event combinations considered (top row) and not considered (bottom row) in the analytical calculations. In all cases, the left crystal represents the source crystal, where the β^−^ decay occurs, and the right crystal is the detector crystal where gamma rays are detected. In Fig. 2a, the β^−^ decay is followed by the emission of all three γ-rays and their subsequent escape. In this (relatively rare, but possible) example, two γ-rays are detected in the detector crystal and a true coincidence is registered. This event contributes to the coincidence energy spectrum to the photopeak corresponding to the sum of the γ_1_+γ_2_ energies together with the non-shifted ^176^Lu β^−^ spectrum. Figures 2b and c are interesting coincidence events in which two of the isomeric transition products γ_2_ or IC_2_, respectively, and γ_3_ deposit all their energy in the source crystal, while γ_1_ is photoelectrically absorbed in the detector crystal. In both cases, the contribution to the coincidence energy spectrum is due to the shift of the ^176^Lu β^−^ spectrum to a value equal to the sum of the isomeric transition energies 2 and 3 (290 keV) and an increment to the photopeak at the energy of γ_1_ (307 keV).

The event represented in Fig. 2d is one in which all three γ-rays are emitted and detected in the emitting crystal; thus, no coincidence is obtained. Clearly, at least one γ-ray is required to be detected in the opposing crystal to produce a true coincidence event. The cases shown in Fig. 2e and f correspond to (rare, but also possible) events in which a coincidence takes place, but a Compton scattering interaction in any of the crystals (shown in blue) is involved. These event combinations are excluded from the analytical calculations since these would require shifting the ^176^Lu β^−^ spectrum to the value of the exact energy deposited by the Compton electron, depending on the scattering angle. Compton events, in which a scattered photon is also detected photoelectrically in a crystal and, therefore, deposit all of the available energy from the isomeric transitions, are included in the calculations. The complete set of events included in the calculations, together with their probabilities, is tabulated in Tables 1 and 2.

**Table 1:** Absorption probabilities for monoenergetic photons simulated in GATE, with 88, 202 and 307 keV energy values, absorbed in the source crystal (C1) and the detector crystal (C2). Probabilities for C1 do not depend on the separation distances

γ-Ray energy (keV)	C1	C2			
		0 cm	2.5 cm	10 cm	21 cm
307	0.6166	0.2608	0.0966	0.0167	0.0043
202	0.8103	0.3540	0.1564	0.0296	0.0079
88	0.9760	0.3898	0.1731	0.0339	0.0088

**Table 2:** Coincidence detection probabilities for each value of deposited energy obtained from the analytical model due to (a) β^−^ decay followed by its subsequent ^176^Hf isomeric transition particles interacting in the source crystal (C1) and (b) γ-ray absorption in the detector crystal (C2)

a						
	*E*_dep_ (keV)	Interactions (γ-ray absorption or IC) in C1	Crystal separation distance (cm)			
			0	2.5	10	21
	0	-	1.60 × 10^−4^	8.33 × 10^−5^	1.76 × 10^−5^	4.70 × 10^−6^
	88	γ_3_ or IC_3_	3.15 × 10^−2^	1.44 × 10^−2^	2.76 × 10^−3^	7.34 × 10^−4^
	202	γ_2_ or IC_2_	6.59 × 10^−4^	3.04 × 10^−4^	6.01 × 10^−5^	1.57 × 10^−5^
	290	(γ_2_ or IC_2_) and (γ_3_ or IC_3_)	8.39 × 10^−2^	3.11 × 10^−2^	5.37 × 10^−3^	1.38 × 10^−3^
	307	γ_1_	2.20 × 10^−4^	1.10 × 10^−4^	2.27 × 10^−5^	6.03 × 10^−6^
	395	γ_1_ and (γ_3_ or IC_3_)	3.44 × 10^−2^	1.52 × 10^−2^	2.87 × 10^−3^	7.67 × 10^−4^
	509	γ_1_ and (γ_2_ or IC_2_)	7.53 × 10^−4^	3.34 × 10^−4^	6.55 × 10^−5^	1.70 × 10^−5^
	597	γ_1_ and (γ_2_ or IC_2_) and (γ_3_ or IC_3_)	0	0	0	0
b						
	*E*_dep_ (keV)	γ-ray absorption in C2	Crystal separation distance (cm)			
			0	2.5	10	21
	0	-	0	0	0	0
	88	γ_3_	1.23 × 10^−3^	6.05 × 10^−4^	1.25 × 10^−4^	3.26 × 10^−5^
	202	γ_2_	5.03 × 10^−2^	2.38 × 10^−2^	4.65 × 10^−3^	1.25 × 10^−3^
	290	γ_2_ and γ_3_	7.30 × 10^−5^	1.53 × 10^−5^	5.86 × 10^−7^	4.08 × 10^−8^
	307	γ_1_	9.43 × 10^−2^	3.61 × 10^−2^	6.37 × 10^−3^	1.65 × 10^−3^
	395	γ_1_ and γ_3_	1.37 × 10^−4^	2.33 × 10^−5^	8.04 × 10^−7^	5.39 × 10^−8^
	509	γ_1_ and γ_2_	5.59 × 10^−3^	9.15 × 10^−4^	2.99 × 10^−5^	2.06 × 10^−6^
	597	γ_1_ and γ_2_ and γ_3_	8.11 × 10^−6^	5.89 × 10^−7^	3.78 × 10^−9^	6.74 × 10^−11^

*E*_*dep*_ deposited energy

The calculations were performed considering four values of *d*: 0, 2.5, 10 and 21 cm. A separation distance *d* = 0 cm is an extreme case (not realistic for positron imaging applications) included only for completeness, a situation that can also be simulated and measured by positioning two detectors in contact, with each crystal covered in reflector material to prevent scintillation light sharing. Finally, the finite energy resolution of the real detector was incorporated in the calculated energy spectra by convolving the analytical prediction with a variable Gaussian kernel as reported in [26].

### Monte Carlo simulations

We performed Monte Carlo simulations with GATE v8.1 of two square LYSO prisms (1 cm thick) with side length of 57.4 mm. The simulations consisted of two LYSO crystals (density and composition information can be found in [27]) aligned face-to-face and separated at distances *d* = 0, 2.5, 10 and 21 cm.

The simulations can be divided into two sets: (1) simulation of monoenergetic photons generated in only one crystal to obtain the detection probabilities for the analytical model and (2) the complete simulation of both scintillation crystals containing point sources of ^176^Lu, uniformly distributed in their volumes.

#### Simulation of monoenergetic photons in singles mode for the analytical model

Monte Carlo simulations of isotropic point-like sources of monoenergetic photons of energies 88, 202 and 307 keV, uniformly generated inside the source crystal facing the detector crystal, were performed. The probabilities of the gamma rays being detected or escaping the source crystal, for a total of 10^5^ primary photons, were in excellent agreement with those reported previously in [27]. The probabilities of the photons escaping from the source crystal and being detected in the detector crystal were obtained for the four separation distances. The probability values were used in the analytical model to compute the coincidence energy spectra. As explained in the “Analytical calculations” section, those events in which scattered photons escaped either crystal depositing a fraction of the available energy were discarded. Compton scattered photons which in turn interacted via photoelectric effect, and therefore deposited all the initial isomeric transition energy, were included.

#### Complete simulation of two LYSO crystals in coincidence mode

The intrinsic radiation of each LYSO crystal was simulated with the ^176^Lu ion source defined in GATE v8.1. Point sources with isotropic emission and a half-life of 3.76 × 10^10^ years were evenly distributed within the crystals simulating an activity concentration of 300 Bq/cm^3^. The total number of simulated decays per crystal and per simulation was approximately 9 × 10^5^.

All the relevant physical processes for photons and electrons interactions (i.e., photoelectric effect, Compton scattering and electron ionization) were included using the GEANT4 standard model, while the β^−^ decay, the gamma ray emission and the internal conversion were incorporated by adding the radioactive decay process in GATE using the default lower limit for electron step size of 0.1 mm. The optical processes of light emission and transport were not included since these drastically increase the simulation time.

The detection in coincidence mode was implemented using the GATE digitizer, which emulates the electronics of the real detector with a coincidence window of 10 ns and considers all coincidence events (“takeAllGoods”) including multiple coincidences. In all the simulations, an 80-keV lower energy threshold was set, a value that corresponds to the energy threshold of the experimental setup. No energy resolution was included at this point; instead, the effects of the scintillation light transport within the crystals, and therefore, the energy resolution was taken into account by convolving the simulated spectra with a variable Gaussian function with standard deviation: *σ*(*E*) = 1.15*E*^0.52^ keV; as formerly obtained experimentally [26]. All spectra were rebinned to 1-keV bins and normalized by the area.

### Experiment

**Fig. 3:**
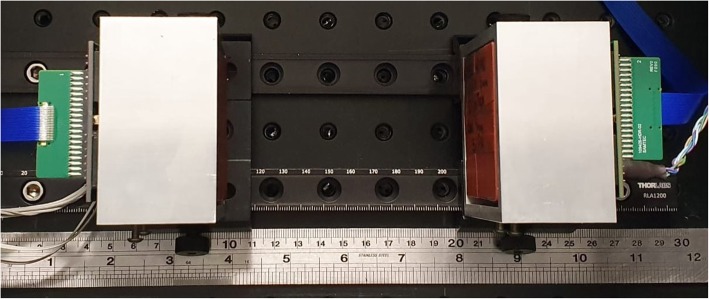
Two detector modules, each consisting of a monolithic LYSO scintillation crystal, wrapped in white Teflon and brown tape. The crystals are coupled to SiPM arrays, SensL ArrayC-60035-64P, and mounted on aluminum cases, which in turn sit on a pair of optical rails to vary the detector separation distance

Two detector modules were aligned face-to-face, each held by an aluminum frame and separated by distances *d* = 0, 2.5, 10 and 21 cm with the aid of an optical rail (Fig. 3). Our detector modules consist of a monolithic square LYSO (Proteus Inc., Chagrin Falls, OH, USA) prism crystal (57.4-mm side length) with all the surfaces polished and wrapped on five sides with white Teflon tape. Each LYSO crystal was coupled to a SiPM ArrayC-60035-64P (SensL Technologies Ltd. Cork, Ireland) using a 6-mm polymethyl-methacrylate (PMMA) light guide and a 1-mm-thick EJ-560 optical interface sheet (Eljen Technology Sweetwater, Texas, USA). The measurements were performed in a light-tight box, and the room temperature was kept at 23 ± 1 °C with air conditioning, with no additional cooling system. Signal readout and processing details can be found in [28].

Acquisitions in coincidence mode due to the intrinsic radioactivity of the crystals (no external sources) were carried out using a time window of 10 ns. The measured timing resolution of our detector arrangement is 1.5 ns. The acquisitions were performed so that 40,000 coincidence events were registered regardless of the separation distance. For energy calibration purposes, the 202 and 307 keV gamma rays, from the isomeric transitions of the^176^Hf excited states, were used. Additionally, a sealed ^22^Na source was used to obtain a third calibration point (511 keV). Finally, all experimental energy spectra were normalized to have an area under the curve equal to unity to allow an easy comparison with the analytical and simulation results.

## Results

### Analytical calculations

The absorption probabilities for the monoenergetic gammas simulated in GATE are listed in Table 1. As mentioned above, the probabilities for 88, 202 and 307 keV gamma rays being absorbed within the source crystal, here labeled as C1 (Column 2), are consistent with previous results reported in [27]. Absorbed gamma rays refer to those gammas that deposited all their energy either by a single photoelectric interaction or by Compton scatterings, ending with a photoelectric interaction, within one crystal. In the following columns (3–6), the probabilities for the gamma rays absorbed in the twin crystal, the one acting as the detector (labeled as C2), are shown as a function of *d*. These probabilities were calculated as the fraction of the number of gammas interacting in C2 with respect to the number of gammas escaping C1.

As expected, the absorption probabilities in C2 decrease for all energy values as *d* increases. Note that the absorption probabilities for C1 are the same for all distances (they do not depend on *d*), since C1 was always the source of the monoenergetic gammas.

Table 2 shows the probabilities used in the analytical model for the calculation of the intrinsic coincidence energy spectra. The values are the result of the combined absorption probabilities of one or two gammas/IC electrons in C1, the source crystal, *and* the full absorption of one, two or three gammas rays in C2, the detector crystal. The values in this table represent the probability of the total energy deposited for each combination in each crystal. For example, the situation in which a full absorption of γ_2_+γ_3_ depositing 290 keV in C1 has to be accompanied with the coincidence detection of γ_1_ depositing 307 keV in C2. Since the absorption of the three gammas (γ_1_+γ_2_+γ_3_) in C1 (i.e., no gamma escapes the source crystal) does not produce a coincidence event, this particular combination has a zero probability (C1, last row, all distances).

Thus, the analytical model involves 4 main steps:

- MC simulation of monoenergetic γ-rays to obtain the absorption probabilities in the source and detector crystals (values in Table 1)
- The isomeric transition (γ-ray or IC electron emission) percentages from the ^176^Lu decay scheme (Fig. 1a) and the γ-ray absorption probabilities (Table 1), considering all the possible combinations that give rise to a true coincidence event, are used to calculate the combined probabilities of Table 2.
- Values from Table 2 are used to normalize the area under the curve of the shifted ^176^Lu beta distributions for each beta and γ-ray detection in the emitting crystal (Table 2 (a)).
- The total energy spectrum is obtained by adding the shifted and normalized beta spectra (Table 2 (a)) in the source crystal and the absorption probabilities of γ-rays in the detector crystal (Table 2 (b)).

It is important to point out that contrary to the analytic calculations reported by Alva-Sánchez et al. [26], the sum of the probabilities for the coincidence spectra calculations does not add up to unity. The reason for this is that the analytical calculations in this work consider only those events in which a true coincidence is involved. Other event types, that do contribute to the total probability, are not included. For example, a 307-keV gamma ray emitted in C1 (and IC for the other two transitions) not interacting with C2 and thus not producing a coincidence event has a certain chance of occurrence not accounted for in the calculations. Additionally, the sum of the probability values decreases with increasing *d*, in view of a smaller chance of any gamma ray emitted in C1 interacting in the distant C2.

**Fig. 4.**
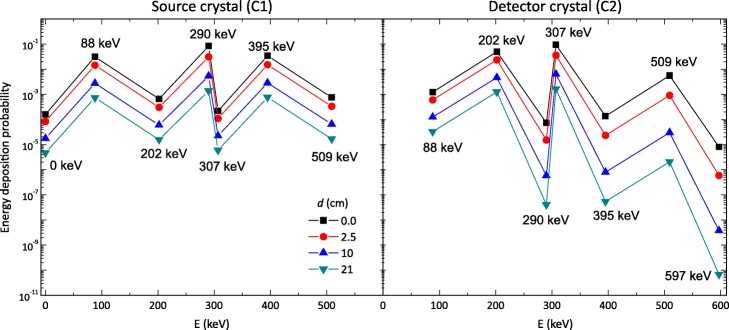
Energy deposition probability values (from Table 2) for the coincidence detection of a β^−^ particle and gamma rays/IC electrons in the source crystal (C1), and one or more gamma rays with energies of 88, 202 and 307 keV in the detector crystal (C2) for four detector separation distances. Probability values equal to zero are not included in the graphs

**Fig. 5:**
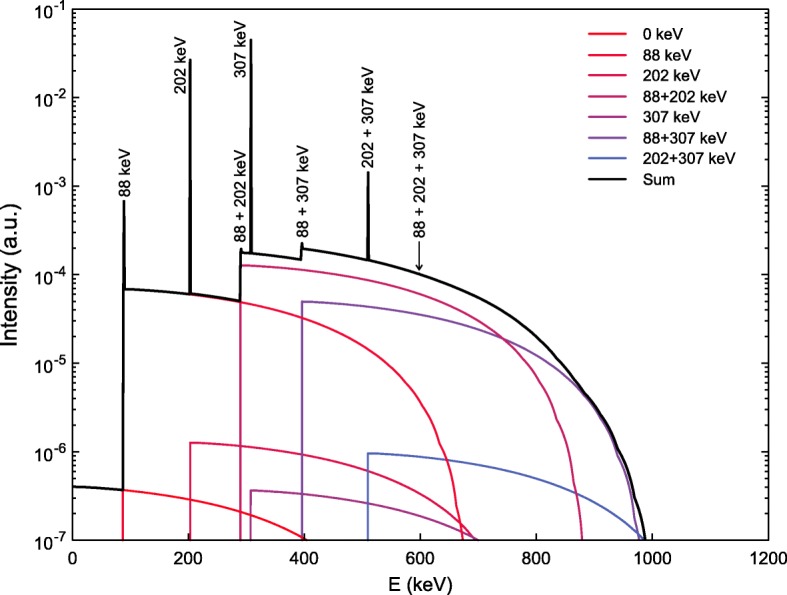
Coincidence energy spectra as predicted by the analytical calculations for two detectors with *d* = 2.5 cm. The different distributions in color are the resulting β^−^ energy spectrum shifted and normalized to the corresponding energy value for each possible interaction combination. The black line is the resulting energy spectrum from summing the β^−^ distributions from the source crystal and the γ-rays from the isomeric transition interactions in the detector crystal. The energy value of the peaks arising from the detection of one, two, or three γ-rays are indicated in the figure (see text for details)

Figure 4 is a plot in a log scale of the probability values of Table 2, offering a more visual understanding of the shape of the coincidence energy spectrum. Bear in mind that there is a difference in several orders of magnitude between the absorption probabilities for the different possible combinations. Consider, for instance, the probability of the 202 and 307 keV gamma rays being detected *individually* in C2. These have relatively very high values, for all separation distances, compared to other combinations, thus explaining the two prominent peaks observed in the experimental spectra. If we look closely at the values for C1, the most probable combinations are those of 88 keV (γ_3_), 290 keV (γ_2_+γ_3_), and 395 keV (γ_1_+γ_3_), explaining the high intensity of the shifted beta spectra in Fig. 5.

Figure 5 shows in different colors the set of beta spectra, shifted to the corresponding energy values and normalized, to reflect the probability for each possible combination, for *d* = 2.5 cm. The black line represents the total coincidence energy spectrum, which is the result of summing the shifted/normalized beta spectra (C1) and the photopeaks (C2). The intensity of the photopeaks also reflects the probability of the gamma rays being detected individually or the simultaneous detection (energy sum) of two or all three gamma rays. Due to the large differences in relative intensities, the vertical axis was plotted in a log scale to be able to visualize all the shifted beta energy spectra. In a linear scale (figure not shown), only the prominent photopeaks are visible. To compare the predictions with the experimental data, the detector resolution needs to be incorporated by the convolution of the final spectra with a variable Gaussian function as explained in the “Complete simulation of two LYSO crystals in coincidence mode” section.

The maximum energy of the true coincidence energy spectra is equal to ${E}_{\beta_{\mathrm{max}}^{-}}+202+307$ keV = 593 + 202 + 307 keV = 1102 keV. This end-point energy is not visible in Fig. 5, since it is at least two orders of magnitude below the end-point energy of the more probable combination corresponding to ${E}_{\beta_{\mathrm{max}}^{-}}+88+307$ keV = 593 + 88 + 307 keV = 988 keV visible in Fig. 5. In the experiment, however, it is possible to have random coincidences (a simultaneous β^−^ decay in each crystal) and the detection of all three gamma rays leading to a possible maximum energy of *E*_max_ = ${E}_{\beta_{\mathrm{max}}^{-}}+88+202+307$ keV = 593 + 88 + 202 + 307 keV = 1190 keV (equal to the *Q*-value between the ground states of ^176^Lu and ^176^Hf). This unlikely, but possible, combination was not included in the analytical calculations and has a negligible effect on the final prediction as is evident when the energy spectra are compared to the experimental data and Monte Carlo simulations, as shown in the following section.

### Analytical vs Monte Carlo simulations and experimental results

**Fig. 6:**
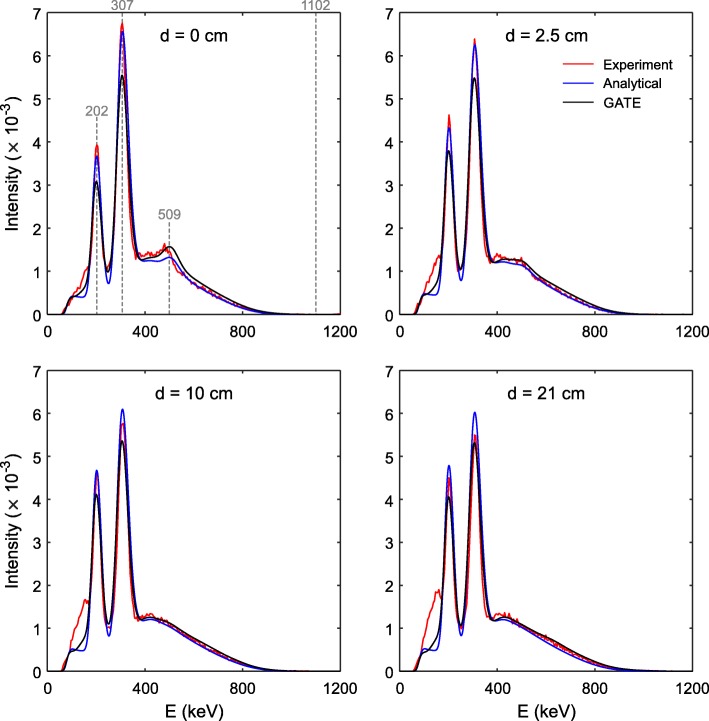
Normalized coincidence energy spectra of two LYSO crystals (57.4 side length) as measured experimentally (red), simulated with GATE (black), and calculated analytically (blue) for four crystal separation distances

Figure 6 shows the normalized coincidence energy spectra for four crystal separation distances as measured experimentally and obtained with the simulations and analytical calculations. There is a remarkable agreement of the gamma ray photopeaks and also, even though the spectra were normalized to have the same area under the curve, all spectra show the same falling edge at high energy values. This confirms that the analytical calculations are able to account for the correct structure of the coincidence spectra as the result of mainly the detection of the 202 and 307 keV gamma rays in the detector crystal (C2), mounted on a continuous distribution due to the simultaneous detection of the beta particles and ^176^Hf isomeric transition products in the source crystal (C1). Certainly, in the experimental setup and Monte Carlo simulations both crystals serve as the source and detector crystals.

Looking closely at the spectra shown in Fig. 6, it is possible to observe that the relative difference in intensity of the 202 and 307 keV peaks decreases with increasing *d*. Experiment, GATE simulations and analytical calculations all show the same tendency: the quotient values between the maximum intensity of the 202 keV photopeak (${I}_{\mathrm{max}}^{202\ \mathrm{keV}}$) with respect to ${I}_{\mathrm{max}}^{307\ \mathrm{keV}}$ agree within 10%. Also, at *d* = 0, there is a small but visible protuberance at 509 keV. This is seen experimentally in the simulation and in the analytical model; the latter explains this as a relatively high probability of the detection of 202 and 307 keV (γ_1_+γ_2_) gamma rays in the detector crystal (C2).

Another visible structure seen in the experimental coincidence energy spectra, but *not* in the Monte Carlo simulations or analytical spectra, is a distribution to the left (at lower energy values) of the 202 keV peak. This structure is more evident at larger separation distances (clearly visible at *d* ≥ 10 cm) and it is probably the result of Compton scattering interactions of the gamma rays with other surrounding objects present in the experiment (e.g., aluminum case, breadboard, SiPM) included neither in the analytical calculations nor in the GATE simulations. For smaller crystal separation distances, the relative contribution of these scattered events to the coincidence energy spectrum is lower compared to larger separation distances.

**Fig. 7:**
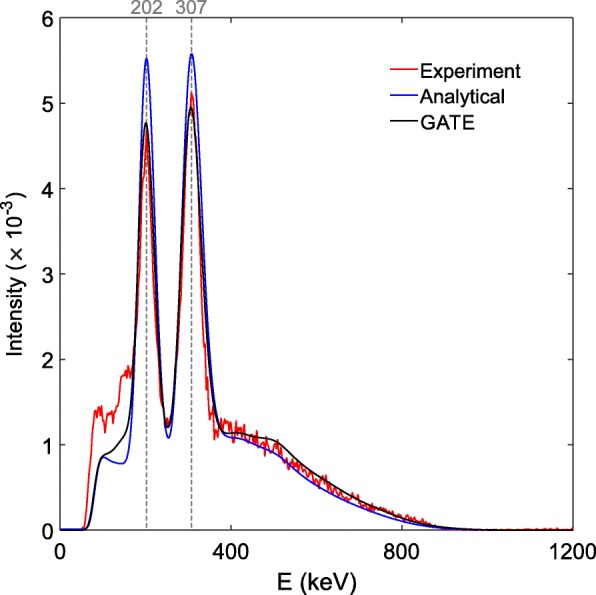
Normalized coincidence energy spectra for two small LYSO cubes (10-mm side length) in contact (*d* = 0 cm) measured experimentally (red), obtained analytically (blue), and simulated in GATE (black)

To test the analytical algorithm, calculations were performed for two small LYSO crystals (10 × 10 × 10 mm^3^) in coincidence mode with *d* = 0 cm, and compared to a Monte Carlo simulation and experimental results. The results are plotted together in Fig. 7. A structure to the left of the 202 keV is also visible in the experimental spectrum, once again attributed to Compton scattering events taking place on the crystals surrounding materials. Still, there is a good agreement between the position of the photopeaks and the relative intensity of the continuous distribution. In contrast to the large crystal, in which the analytical model shows an excellent agreement with the experimental spectra, in this case, the accordance between the analytical calculations and the experiment is somewhat lower. However, the GATE simulation shows a better agreement with the experiment, a fact that can be explained by a higher probability for Compton scattered photons to escape from the small crystal than from the large one, precisely the type of events included in the simulations but not in the analytical model.

## Discussion

### Analytical model vs experiment and simulations

The concordance between the analytical model, the experiment and the Monte Carlo simulations, although remarkable, is not perfect due to a number of probable reasons. Recall that the analytical model does not include events in which scattered photons escaped from either crystal depositing only a fraction of their available energy. Both the analytical model and Monte Carlo simulations consider only the ionizing radiation transport and its detection probability in the crystal. They do not include the light transport in the crystal: for the experiment we used white Teflon as the scintillator wrapping material, but other reflectors may produce a fair degree of differences. The response of the photodetector and electronics is also not included, and the energy resolution was accounted for through the convolution of the predicted energy spectra with a variable Gaussian kernel that reflects the measured energy resolution. For the Monte Carlo simulations using GATE, our results are in good agreement with those reported in [22, 24].

Experimentally, acquisitions in coincidence mode require a time window for “simultaneous” events to be registered. Note, however, that the calculations do not take account of time; the analytical spectra are the result of the combination of emission and absorption probabilities, which depend on the crystal size and separation distance. In real scenarios, coincidence energy spectra could be modified by the width of the coincidence time window due to the detection of random coincidences, with a bigger impact when using wider time windows. Moreover, the number of random coincidences will depend on the detector configuration: more random events can be expected in organ-specific imaging probes, where the opposing detectors can be positioned closer to each other. For example, beta decay may occur in one crystal and, at the same time, a gamma ray (emitted from a different crystal) is detected in the opposing detector generating a random coincidence event.

### Coincidence counting rates

**Fig. 8:**
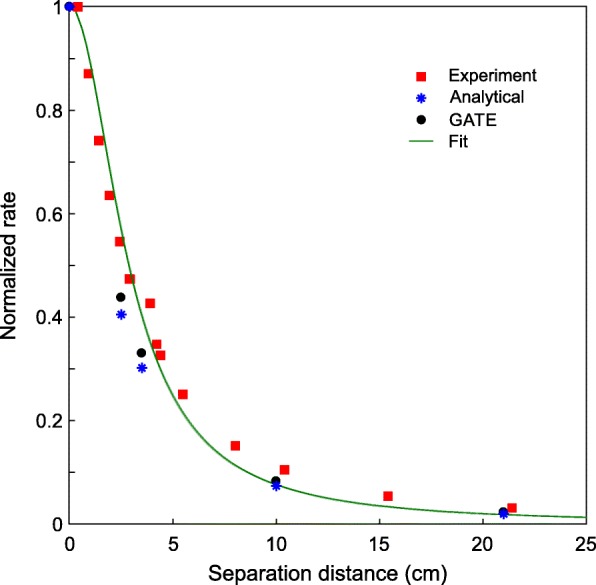
Normalized count rate for two opposing LYSO scintillation crystals of dimensions 57.4 × 57.4 × 10 mm^3^ as a function of *d*. Note that for the experimental count rate more separation distances were included for completeness. Experimental uncertainties for the detector separation distance are smaller than the symbol size.

Figure 8 shows the normalized coincidence count rate for the experiments and the GATE simulations as a function of *d*. The sum of the absorption probabilities in C2 or C1 from Table 2 (normalized by the maximum value at *d* = 0) are also plotted in the same figure. In the experiment and in the complete Monte Carlo simulations, both the solid angle between the crystals and the coincidence time window are involved in the coincidence count rates. On the other hand, although in the analytical model no timing is included, the absorption probabilities as a function of *d* reflect the same trend as the count rate. Once more, the agreement is not entirely perfect since the analytical model does not include Compton scattered events escaping from the crystals. Nevertheless, the model can be used to anticipate the coincidence count rate considering the detector disposition, especially when the random coincidence rate is low.

A fit (green continuous line) to the experimental counting rate as a function *d* was carried out of the form:

$$ f(d)=\frac{1}{1+{cd}^2}, $$

with *c* = 0.1212 as the fitted constant parameter. The good fit (*R*^2^ = 0.9814 and sum squared error = 0.0225) of this function is in agreement with the expected 1/*d*^2^ dependence for large distances, while at *d* = 0 a finite rate value is obtained. For other crystal sizes and geometries, as in a complete ring of detectors, the coincidence counting rate will be different.

The experimental count rate for random coincidences, i.e., the simultaneous detection of two β^−^ decays in both crystals, was around 4 counts per second (cps) for the large crystals and was obtained by locating the detectors at a large distance (*d* > 21 cm) in a position such that gamma rays from one detector were very unlikely to reach the other. This very low counting rate for random coincidences, observed in the experiment, explains why the analytical model correctly predicts energy measured coincidence energy spectra for all *d* values.

### Transmission scans and detector calibration

As pointed out by Rothfuss et al. [15], transmission scans to produce attenuation correction maps using the 202 and 307 keV gamma rays in coincidence mode are possible as long as (a) the energy windows are appropriately defined, (b) the coincidence time window is increased to allow for their detection in coincidence mode (especially in scanners incorporating TOF with ≤ 4 ns timing windows), and (c) the linear attenuation coefficients are scaled-up to 511 keV (easier for monoenergetic photons vs the continuous x-ray spectrum in computed tomography). Given the low coincidence rates due to the intrinsic radioactivity, the transmission scans can be more useful in scenarios involving organ-specific imaging where the detectors can be placed close to each other. The model developed in this work allows predicting the proportion of the detected 202 and 307 keV gamma rays depending on the detector separation, important for transmission scans.

Given the long half-life of ^176^Lu, the results of this work make the intrinsic radioactivity of LYSO scintillation crystals very convenient for detector calibration and quality control procedures. Thus, the full understanding and correct application of this phenomenon can potentially substitute the ^68^Ge/^68^Ga external sources currently used for this purpose, if a sufficiently long acquisition is performed. The usefulness of this method could be even more beneficial in preclinical or organ-dedicated systems.

## Conclusions

In spite of the exclusion of the events in which scattered photons manage to escape from the crystal, the analytical calculations correctly reproduce the general shape of the coincidence energy spectrum obtained experimentally and with Monte Carlo simulations using GATE. The analytical model is a fast and robust method that provides a clear understanding, from the physics point of view, of the processes and their contributions producing the energy spectra arising from the LYSO intrinsic radioactivity in coincidence mode. Following the procedure described in this work, our method can be extended to include a more complex geometry, as in the set of detectors comprising a PET scanner, the two detector panels of a positron emission mammography system, or any other positron imaging system, to predict the contribution of the background signal of the crystals if the exact size and geometry of the detector crystals is known.

For Monte Carlo simulations of PET systems, this work can also be used as a starting point to check that a particular code correctly models the background signal from radioactive detectors. Particularly, for the GATE code, our methods reproduce with good agreement the intrinsic coincidence energy spectra of LYSO crystals without the inclusion of the crystal light emission and transport, which would in turn increase the computation time and the complexity of the parameters to be defined in the simulation.

As in the case of understanding the shape of the energy spectrum in singles mode, this work provides all the details necessary involved in the structure of the coincidence energy spectrum. This could translate into an effective opportunity for detector calibration in coincidence mode and may prove very valuable for research teams developing transmission imaging techniques using the natural radioactivity of lutetium-based scintillation detectors.

## Data Availability

The datasets used and/or analyzed during the current study are available from the corresponding author on reasonable request.

## Abbreviations

*E*_dep_: Deposited energy
GATE: GEANT4 Application for Tomographic Emission
IC: Internal conversion
LSO: Cerium-doped lutetium oxyorthosilicate
LYSO: Cerium-doped lutetium yttrium oxyorthosilicate
PET: Positron emission tomography
TOF: Time-of-Flight
